# Assessment of spontaneous resolution of idiopathic bone cavity

**DOI:** 10.1590/1678-7757-2017-0288

**Published:** 2018-04-18

**Authors:** Maíra de Paula Leite Battisti, Mariana Quirino Silveira Soares, Cássia Maria Fischer Rubira, Izabel Regina Fischer Rubira de Bullen, José Roberto Pereira Lauris, José Humberto Damante

**Affiliations:** 1 Universidade de São Paulo, Faculdade de Odontologia de Bauru, Departamento de Cirurgia, Estomatologia, Patologia e Radiologia, Bauru, São Paulo, Brasil.; 2 Universidade de São Paulo, Faculdade de Odontologia de Bauru, Departamento de Odontopediatria, Ortodontia e Saúde Coletiva, Bauru, São Paulo, Brasil.

**Keywords:** Bone cysts, Solitary cyst, Bone remodeling, Panoramic radiography, X-rays

## Abstract

Idiopathic Bone Cavity (IBC) or Simple Bone Cyst (SBC) is a non- epithelialized bone cavity with serosanguinous fluid content or empty. There is a literature debate regarding its pathogenesis that remains unclear. The main treatment option is the surgical exploration, although there are successful cases described in the literature in which just a follow-up with clinical and radiographic evaluation was performed.

**Objective:**

This study aimed to assess the spontaneous resolution of idiopathic bone cavity untreated by surgery.

**Material and Methods:**

Twenty-one patients diagnosed with surgically untreated IBC were submitted to a follow-up protocol modified from Damante, Guerra, and Ferreira^5^ (2002). A clinical and radiographic evaluation was performed in 13 patients (13/21), while eight patients (8/21) were only radiographically evaluated. Three observers evaluated the panoramic radiographs of 21 patients and the Kappa test was performed by intra and inter-examiners. Inductive and descriptive statistics were applied to the results.

**Results:**

Only one patient had a positive response to palpation and percussion of the teeth in the cyst area. Most of the cysts evaluated were rated as 3 (lesion “in involution”), 4 (lesion “almost completely resolved”), or 5 (“completely resolved”).

**Conclusions:**

We observed progressive spontaneous resolution of IBC. Most cysts were found in the recovery process in different follow-up periods. Patient's follow-up, without surgery, may be considered after the diagnosis based on epidemiological, clinical, and radiographic features of the lesion.

## Introduction

Idiopathic Bone Cavity (IBC), Traumatic Bone Cyst, or Simple Bone Cyst (SBC) is a commonly asymptomatic intraosseous cavity not covered by epithelium, empty or partially filled with serous/bloody fluid. It has a thin membrane of connective tissue, only microscopically visible, overlying the bone surface. It is almost totally located in the cancellous bone of the lower jaw, most in the premolars and molars region^8^ ^,^ ^15^ ^,^ ^16^ ^,^ ^22^. A supposed etiology may be linked to a hemorrhagic or ischemic vascular phenomenon with subsequent bone necrosis and resorption^9^. This lesion affects young people between the 1^st^ and 2^nd^ decades of life^8^. The IBC image appears as a radiolucent limited area, usually oval or circular shaped, partially limited by a well-defined, sometimes radiopaque, line. Lesion boundaries are usually below the roots and may be superimposed to - or bypass - the roots. Extension of the crest involvement presents a “scalloping” aspect. The body of the mandible is the most frequently affected area^8^ ^,^ ^12^ ^,^ ^16^.

Clinically, the alveolar ridge is covered by normal oral mucosa, and the expansion of buccal and lingual cortex are rare. The teeth involved are vital, with rare displacement^8^ ^,^ ^12^. Besides the panoramic radiograph, occlusal and periapical X-rays are sometimes required. Cone beam computed tomography (CBCT) may be prescribed in some cases^14^.

IBC are mostly incidentally found in radiographic examinations performed for other reasons, especially when prescribed in the assessment of an orthodontic patient^8^ ^,^ ^5^ ^,^ ^12^ ^,^ ^13^ ^,^ ^19^.

Few studies described cases of untreated IBC with spontaneous resolution, highlighting possible non-surgical treatment for IBC cases, reducing risks of morbidity^9^ ^,^ ^20^ ^,^ ^22^ ^,^ ^23^. This study aimed to assess the spontaneous resolution of surgically untreated IBC cases by panoramic radiographs and CBCT images.

## Material and methods

After approval of this research by the Ethics Committee for Human Research (15275413.1.0000.5417), 21 patients diagnosed with surgically untreated IBC were recalled and submitted to clinical and radiographic examinations for medical follow-up. Only 13 patients (13/21) showed up for the examinations, and the other eight (8/21) had their archived radiographs analyzed.

The follow-up periods were different in the 21 cases, thus, they were divided into 3 groups: Group A – 1 to 5 years; Group B - from 6 to 10 years; and Group C - over 10 years of follow-up.

Examinations of the oral cavity of 13 patients were performed through a systematic evaluation of the integrity of its hard and soft tissues and presence or symptoms of cortical expansion. Panoramic radiographs were prescribed for all. CBCT was also indicated in only one case. All the criteria for X-ray prescription and protection of patients were observed.

The recent panoramic image obtained from the 21 patients was analyzed and compared to the initial panoramic image. The lesions were classified (slightly adapted) according to Damante, Guerra, and Ferreira^5^ (2002):

1. Lesion “in evolution,” when there was an increase in the extent of the radiolucent image changing the mesio-distal relationship, considering the roots of the teeth as a parameter.
2. “Static” lesion, when the shape and size of the radiolucent area remained equal or almost equal to the initial image.
3. Lesion “in involution,” when there was a decrease of the above mentioned dimensions and/or appearance of trabecular bone in the radiolucent area.
4. Lesion “almost completely resolved,” when there was incomplete disappearance of the pathological area, such as traces of the margins that allowed to identify the lesion, but its interior was already remodeled.
5. Lesion “completely resolved,” when there was complete disappearance of the lesion without any radiographic sign.

Three examiners performed the evaluation of the initial and final panoramic image. Two of them were oral radiology specialists (Examiners A and B) and the third was a general practitioner without expertise in the area (Examiner C).

All examiners were trained according to an interpretation guide. They performed a pilot analysis of 30% of the sample and, after 15 days, the entire sample (21 patients). Inter-reviewer reliability was analyzed by calculating percentage agreement and a Kappa test (κ).

## Results

### Clinical evaluation

Twenty-one cases of untreated IBC were requested to a clinical evaluation. Clinical data regarding all patients included in this study is presented on Table 1. Only 13 of them attended to the Oral Medicine Service. Twelve had normal oral conditions with no symptoms and no expansion of cortical bone. One case had slight pain and teeth sensibility close to the lesion. The patient had occlusion problems that may explain the pain complaint. The teeth were vital and suffered no trauma, thus, the sensitivity was classified as a possible dysesthesia of psychogenic origin by the patient's knowledge regarding the existence of IBC in his mandible.

**Table 1:** Clinical data of the patients

Description		N (%)
Gender	Female	13 (61.90%)
	Male	8 (38.09%)
Age at the moment of the diagnosis	2^nd^ decade of life	19 (90.47%)
	3^nd^ decade of life	2 (9.53%)
Number of lesions	Single	19 (40.47%)
	Bilateral	2 (9.53%)
Location of the lesions (n=23)	Anterior	9 (39.13%)
	Posterior	14 (60.87%)
Total of patients		21 (100%)

### Panoramic evaluation

The intra-examiner agreement (concerning the bone remodeling stage of the lesion) was satisfactory (“substantial agreement”), whereas the intra-examiner Kappa test was 0.8 for Examiner A; 1.0 for Examiner B, and 0.75 for Examiner C. The concordance was also considered good, with a Kappa value of 0.75.

**Figure 1:**
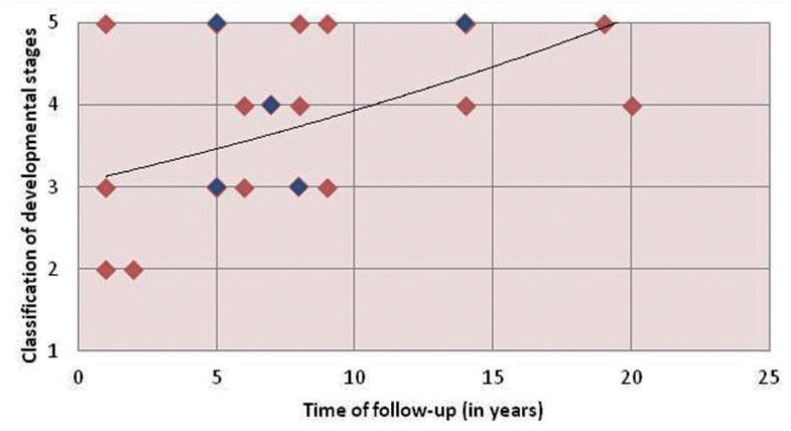
Distribution of idiopathic bone cavity (IBC) lesions in accordance with classification of developmental stage and follow-up period* *The blue dots represent coincidences and each blue dot represents 2 lesions. There are a total of 23 injuries because 2 cases were bilateral

**Figure 2:**
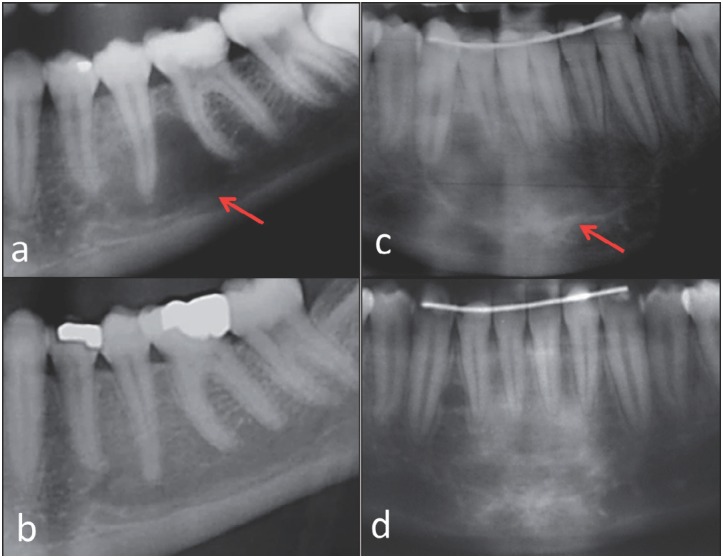
Radiographs showing spontaneous resolution of idiopathic bone cavity (IBC). a: A 15-year old female displaying an unilocular, well-defined radiolucency in the posterior mandible (red arrow); b: Final panoramic radiograph of the same patient after eight years of follow-up, showing complete resolution of the lesion; c: A 15-year-old female displaying an unilocular, well-defined radiolucency with scalloped margin surrounding the teeth apex in the anterior mandible (red arrow); d: After eight years of follow-up, we observed regression of the lesion

There was a higher tendency of the cases to be classified as 3, 4, or 5 according to the follow-up period in years (Group A, B, or C). However, three cases from Group A (1 to 5 years) were classified as stage 5 (“completely resolved”). Most of the lesions were classified as stages 3, 4, and 5 within the observation period of 5 to 10 (Figure 1; Figure 2).

## Discussion

The IBC is a benign lesion with unknown pathogenesis and etiology^9^. Since it was first described by Lucas^11^ (1929), several etiological hypotheses have emerged, remaining a matter of great discussion. According to Harnet, et al.^9^ (2008), three theories predominate: the first one points a local abnormality during bone growth as responsible for the IBC formation. This theory might explain its main location in the region near the mental foramen, where the primary ossification points of the mandible are located^9^. The second theory raises the hypothesis that IBC is part of a tumor process. This theory is based on clinical observations where, in some cases, the IBC is associated to fibrous dysplasia^7^. The conversion of IBC into a central giant cell granuloma immediately after surgical intervention was also reported^3^. Accordingly to the third hypothesis, a low intensity trauma that would induce intramedullary hemorrhage and hematoma or even ischemia and necrosis could lead to IBC lesion formation^9^. However, this hypothesis is contested by the absence of history of local trauma in several cases^12^.

The debate about IBC etiology is also reflected by the extensive nomenclature found in the literature. Different terms have been used as synonyms to refer to this lesion, attempting to reflect its origin and radiographic and clinical presentation. Among them: simple bone cyst, traumatic bone cyst, unicameral bone cyst, solitary bone cyst, hemorrhagic bone cyst, extravasation cyst, and progressive bone cavity^2^ ^,^ ^4^ ^,^ ^9^ ^,^ ^13^ ^,^ ^15^ ^−^ ^17^ ^,^ ^22^.

According to Shear and Speight^22^ (2007), surgical exploration is the most recommended treatment, however, the possibility of spontaneous resolution after an adequate clinical and radiographic examination is also mentioned. Other authors also point out the possibility of spontaneous resolution^9^ ^,^ ^13^.

Blum^1^ described the first case reporting spontaneous IBC resolution in 1955. The author proposed that the nomenclature *progressive bone cavity* should be avoided, since bone regeneration was observed without any intervention in his clinical experience^1^. In 1966, Szerlip^23^ confirmed Blum's observations, reporting another untreated case with spontaneous remodeling after 5 years of follow-up. Sapp and Stark^20^ (1990) reported two more cases of spontaneous resolution. In these cases, the time lapse between diagnosis and resolution was seven years in one case and two years in the other. The authors considered that the high incidence of idiopathic bone cavity in young people and rare occurrence in people over 25 years old reinforced the possibility of spontaneous resolution^23^.

Based on clinical experience on spontaneous regression of IBC, Damante, Guerra, and Ferreira^5^ (2002) suggested a clinical and radiographic followup protocol, presenting 10 cases with spontaneous regression in different time periods. In that study the authors reported complete remission of the lesion in one case. The follow-up period varied between two to seven years with mean of 3.8 years^5^.

This study reports 21 cases submitted to a slight modified protocol established by Damante, Guerra and Ferreira^5^ (2002). In 13 patients clinically evaluated, only one had slightly painful teeth sensitivity not related to the lesion.

Panoramic analyses showed a tendency of the lesions to be classified as stages 3 (lesion “in involution”), 4 (lesion “almost completely resolved”), and 5 (lesion “completely resolved”) (Graph 1). Most cases that were followed from one to 20 years in this study showed that idiopathic bone cavity is a disease which affects children and young people (Table 1). The IBC underwent complete resolution with no intervention in eight cases. Thus far, this is the biggest report of IBC spontaneous resolution. Our results highlight the possibility of non-intervention follow-up for IBC lesions after clinical and radiographic diagnosis.

The surgical approach is still considered a safe procedure for the diagnosis and treatment of IBC^18^ ^,^ ^24^. Surgery must be performed for symptomatic cases whenever boundary changes, or size and cortical expansion occurs. Anytime the IBC lesion presents unusual clinical and radiographic characteristics, surgical exploration must also be performed to confirm the diagnosis and exclude other lesions. Differential diagnosis for IBC must include fibro-osseous lesions, odontogenic keratocyst, ameloblastoma, aneurysmatic bone cyst, central giant cell granuloma, among others^6^ ^,^ ^10^ ^,^ ^21^. The nonsurgical treatment of IBC may not be applied to all cases, but it can be useful to most of the suspicious lesions if the patient is available for long term follow-up.

## Conclusions

The results of this study indicate that a spontaneous resolution of untreated idiopathic bone cavity may occur. The IBC follow-up without surgical intervention is possible after the diagnosis, based on epidemiological, clinical, and radiographic characteristics described in the literature. The long period need for the complete resolution of the lesion, in some cases, must also be considered while electing this protocol.

## References

[1] Blum T (1955). An additional report on traumatic bone cysts; also a discussion of Dr. John G. Whinery's paper, “Progressive Bone Cavities of the Mandible”. Oral Surg Oral Med Oral Pathol..

[2] Bruce KW (1965). Traumatic (extravasation - hemorrhagic) bone cyst of the mandible. Chronicle..

[3] Chiba I, Teh BG, Iizuka T, Fukuda H (2002). Conversion of a traumatic bone cyst into central giant cell granuloma: implications for pathogenesis - a case report. J Oral Maxillofac Surg..

[4] Copete MA, Kawamata A, Langlais RP (1998). Solitary bone cyst of the jaws: radiographic review of 44 cases. Oral Surg Oral Med Oral Pathol Oral Radiol Endod..

[5] Damante JH, Guerra EN, Ferreira O (2002). Spontaneous resolution of simple bone cysts. Dentomaxillofac Radiol..

[6] Ferreira O, Damante JH, Lauris JR (2004). Simple bone cyst versus odontogenic keratocyst: differential diagnosis by digitized panoramic radiography. Dentomaxillofac Radiol..

[7] Fisher AD (1976). Bone cavities in fibro-osseous lesions. Br J Oral Surg..

[8] Hansen LS, Sapone J, Sproat RC (1974). Traumatic bone cysts of jaws. Oral Surg Oral Med Oral Pathol..

[9] Harnet JC, Lombardi T, Klewansky P, Rieger J, Tempe MH, Clavert JM (2008). Solitary bone cyst of the jaws: a review of the etiopathogenic hypotheses. J Oral Maxillofac Surg..

[10] Harris SJ, Carroll MK, Gordy FM (1992). Idiopathic bone cavity (traumatic bone cyst) with the radiographic appearance of a fibro-osseous lesion. Oral Surg Oral Med Oral Pathol..

[11] Lucas CD, Blum T (1929). Do all cysts of the jaws originate from the dental system. J Am Dent Assoc..

[12] Martins PR, Santos TS, Araújo VL, Santos JS, Andrade ES, Silva LC (2012). Traumatic bone cyst of the mandible: a review of 26 cases. Br J Otorhinolaryngology.

[13] Mascard E, Gomez-Brouchet A, Lambot K (2015). Bone cysts: unicameral and aneurysmal bone cyst. Orthop Traumatol Surg Res..

[14] Mathew R, Omami G, Gianoli D, Lurie A (2012). Unusual cone-beam computerized tomography presentation of traumatic (simple) bone cyst: case report and radiographic analysis. Oral Surg Oral Med Oral Pathol Oral Radiol..

[15] Matsumura S, Murakami S, Kakimoto N, Furukawa S, Kishino M, Ishida T (1998). Histopathologic and radiographic findings of the simple bone cyst. Oral Surg Oral Med Oral Pathol Oral Radiol Endod..

[16] Perdigão PF, Silva EC, Sakurai E, Soares de, Araújo N, Gomez RS (2003). Idiopathic bone cavity: a clinical, radiographic, and histological study. Br J Oral Maxillofac Surg..

[17] Reddy GS, Reddy GV, Phanitej G, Reddy A, Priyadarshini S, Reddy K (2016). Hemorrhagic bone cyst of mandible: a case report. International J Case Rep Images..

[18] Resnick CM, Dentino KM, Garza R, Padwa BL (2016). A management strategy for idiopathic bone cavities of the jaws. J Oral Maxillofac Surg..

[19] Sapone J, Hansen LS (1974). Traumatic bone cysts of jaws: diagnosis, treatment, and prognosis. Oral Surg Oral Med Oral Pathol..

[20] Sapp JP, Stark ML (1990). Self-healing traumatic bone cysts. Oral Surg Oral Med Oral Pathol..

[21] Scholl RJ, Kellett HM, Neumann DP, Lurie AG (1999). Cysts and cystic lesions of the mandible: clinical and radiologic-histopathologic review. Radiographics..

[22] Shear M, Speight P (2008). Cysts of the oral and maxillofacial regions.

[23] Szerlip L (1966). Traumatic bone cysts. Resolution without surgery. Oral Surg Oral Med Oral Pathol..

[24] You MS, Kim DY, Ahn KM (2017). Surgical management of idiopathic bone cavity: case series of consecutive 27 patients. J Korean Assoc Oral Maxillofac Surg..

